# Effectiveness of training interventions to improve quality of medical certification of cause of death: systematic review and meta-analysis

**DOI:** 10.1186/s12916-020-01840-2

**Published:** 2020-12-11

**Authors:** U. S. H. Gamage, Pasyodun Koralage Buddhika Mahesh, Jesse Schnall, Lene Mikkelsen, John D. Hart, Hafiz Chowdhury, Hang Li, Deirdre McLaughlin, Alan D. Lopez

**Affiliations:** Melbourne School of Population and Global Health, University of Melbourne, 207 Bouverie street, Melbourne, 3053 Australia

**Keywords:** Medical certification of cause of death, Medical education, In-service medical training, Quality of death certification, Effectiveness of training, Vital registration, Civil registration and vital statistics

## Abstract

**Background:**

Valid cause of death data are essential for health policy formation. The quality of medical certification of cause of death (MCCOD) by physicians directly affects the utility of cause of death data for public policy and hospital management. Whilst training in correct certification has been provided for physicians and medical students, the impact of training is often unknown. This study was conducted to systematically review and meta-analyse the effectiveness of training interventions to improve the quality of MCCOD.

**Methods:**

This review was registered in the International Prospective Register of Systematic Reviews (PROSPERO; Registration ID: CRD42020172547) and followed Preferred Reporting Items for Systematic Reviews and Meta-Analyses (PRISMA) guidelines. CENTRAL, Ovid MEDLINE and Ovid EMBASE databases were searched using pre-defined search strategies covering the eligibility criteria. Studies were selected using four screening questions using the Distiller-SR software. Risk of bias assessments were conducted with GRADE recommendations and ROBINS-I criteria for randomised and non-randomised interventions, respectively. Study selection, data extraction and bias assessments were performed independently by two reviewers with a third reviewer to resolve conflicts. Clinical, methodological and statistical heterogeneity assessments were conducted. Meta-analyses were performed with Review Manager 5.4 software using the ‘generic inverse variance method’ with risk difference as the pooled estimate. A ‘summary of findings’ table was prepared using the ‘GRADEproGDT’ online tool. Sensitivity analyses and narrative synthesis of the findings were also performed.

**Results:**

After de-duplication, 616 articles were identified and 21 subsequently selected for synthesis of findings; four underwent meta-analysis. The meta-analyses indicated that selected training interventions significantly reduced error rates among participants, with pooled risk differences of 15–33%. Robustness was identified with the sensitivity analyses. The findings of the narrative synthesis were similarly suggestive of favourable outcomes for both physicians and medical trainees.

**Conclusions:**

Training physicians in correct certification improves the accuracy and policy utility of cause of death data. Investment in MCCOD training activities should be considered as a key component of strategies to improve vital registration systems given the potential of such training to substantially improve the quality of cause of death data.

**Supplementary Information:**

The online version contains supplementary material available at 10.1186/s12916-020-01840-2.

## Background

The death certificate is a permanent, legal record of death that provides important information about the circumstances and cause of death [1]. For deaths that occur in hospitals, or other settings where a doctor is present, death certification is initiated by a medical officer, after which the certificate usually undergoes registration by a national civil registration system [2].

Accurate and timely cause of death reporting is essential for health policy and research purposes [3]. Individual death certificates are routinely aggregated into vital statistics by national civil registration systems, providing the most widely verified sources of mortality data in the form of standardised, comparable, cause-specific mortality figures [4]. These statistics provide essential insights for government policymakers, health managers, healthcare providers, donors and research institutes into common causes of death by age, sex, location and time. The data inform the allocation of resources across an array of stakeholders and disciplines, including medical research and education, disease control, social welfare and development and health promotion [5].

### Cause of death

The ‘gold standard’ for cause of death statistics is complete civil registration where each death has an underlying cause assigned by a physician and is coded according to International Classification of Diseases (ICD) rules. Causes of death reported in death certificates are defined by the World Health Organization (WHO) as ‘all those diseases, morbid conditions or injuries which either resulted in or contributed to death and the circumstances of the accident or violence which produced any such injuries’ [6]. Importantly, this definition does not include symptoms and modes of dying.

### Medical certification of cause of death

**Fig. 1:**
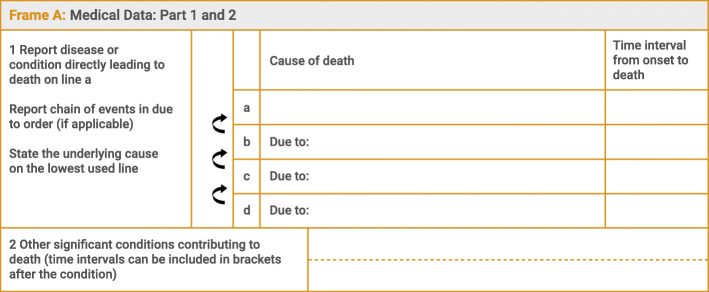
Frame A (Medical data: Part 1 and 2) of the International Form of Medical Certificate of Cause of Death

The Medical Certificate of Cause of Death (Fig. 1) is a standardised universal form recommended by the WHO for international use, which has been adopted by most WHO member states [6]. The WHO also provides instructions on correct cause of death reporting to improve the quality of medical certification and subsequent data [7].

When a single cause of death is reported on the death certificate, this becomes the underlying cause of death used for tabulation. When more than one cause of death is reported, the disease or injury which initiated the sequence of events that produced the fatal event becomes the underlying cause of death [6].

Despite the availability of guidance, errors in cause of death certification have been observed across all geographical regions, with inadequate certification by doctors remaining the principal reason for inaccurate death data [8, 9]. Over the past few decades, therefore, training medical doctors in death certification has become a key intervention employed by health services and national governments to improve mortality statistics. Interventions have included improvements in death certificate formats, training programmes on completion of death certificates, development of self-learning educational materials, implementation of cause of death query systems, periodic peer auditing of death certificates and increasing autopsy rates [10–12].

### Intervention studies on death certification

Several studies have investigated the effectiveness of interventions to improve the quality of death certification [13–15]. Whilst improvement in death certification accuracy is often reported, negative findings have also been published [16]. Moreover, there are few randomised controlled trials (RCTs) or similar studies that have produced high-quality evidence. A 2010 literature review identified 129 studies on the effectiveness of educational interventions for death certification, ultimately reviewing 14, including three RCTs [8]. All educational interventions identified in the review improved certain aspects of death certification, although the statistical significance of evaluation results varied with the type of intervention.

Given the absence of any systematic review and meta-analysis of death certification training interventions, as well as the increase in experimental data produced in the past decade and the need—made even more urgent by the COVID-19 pandemic—to strengthen national vital registration and cause of death data systems, further evaluation is essential. In this study, we systematically review and meta-analyse the effectiveness of training interventions for improving the quality of medical certification of cause of death (MCCOD). To our knowledge, no study has specifically investigated interventions intended to reduce errors in MCCOD in a systematic review.

## Methods

### Preparation and search strategy

This review was registered in the International Prospective Register of Systematic Reviews (PROSPERO; Registration ID: CRD42020172547). Preferred Reporting Items for Systematic Reviews and Meta-Analyses (PRISMA) guidelines were followed throughout the review process [17].

A comprehensive literature search was conducted to identify published articles investigating the effectiveness of training and education interventions to improve death certification (additional file 1: Fig. S1). The search was conducted on the CENTRAL, Ovid MEDLINE and Ovid EMBASE electronic databases, and returned 1060 results, which were exported to EndNote X9 citation manager and deduplicated. The remaining 676 studies were then limited to those published from 1994 onwards (where 1994 is the year ICD-10 was implemented) resulting in 616 studies for screening.

### Eligibility criteria and study selection

**Fig. 2:**
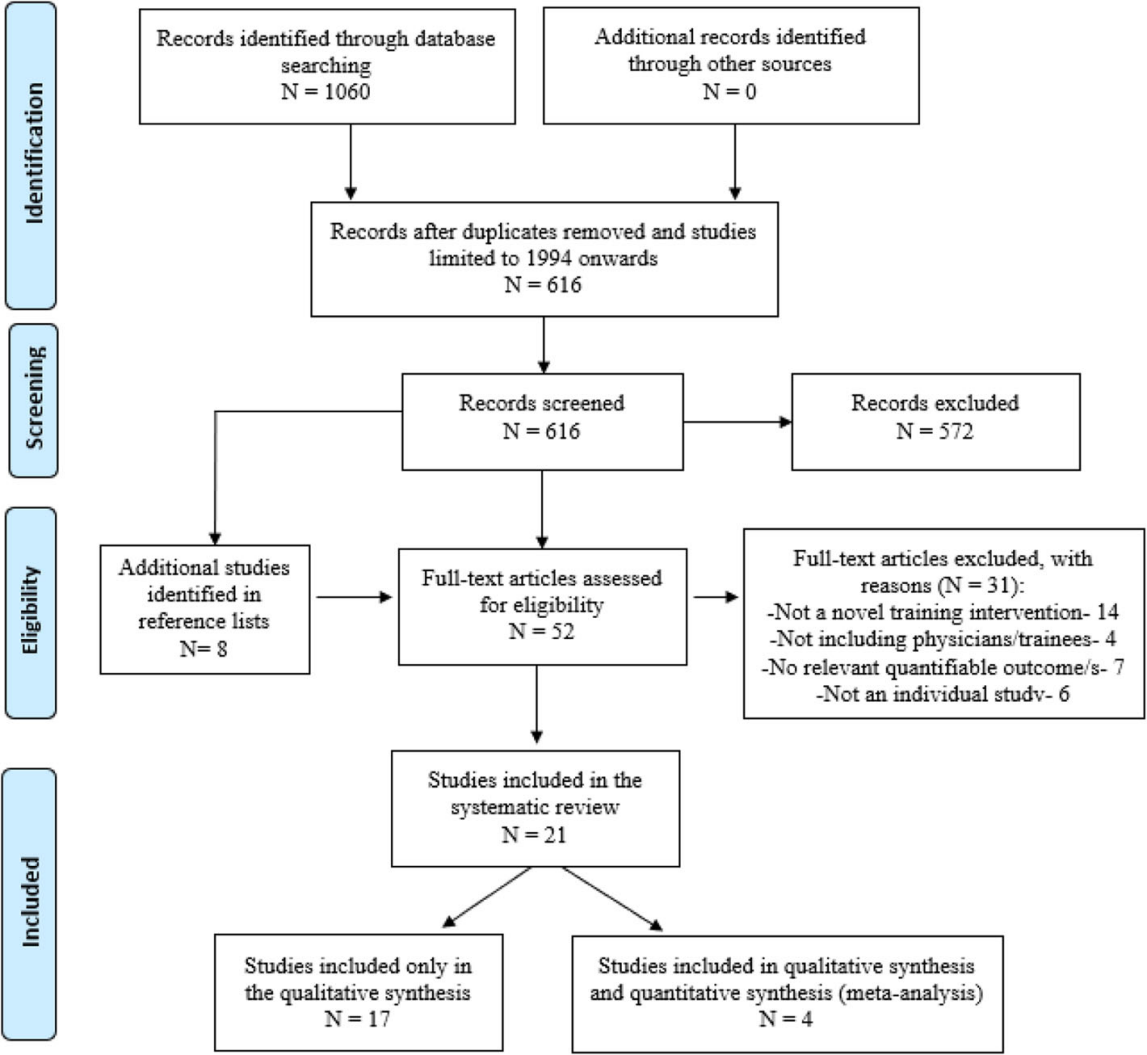
PRISMA flow diagram

This study aimed to assess the effectiveness of training interventions in improving the quality of MCCOD compared to generic academic training in training curricula for current, as well as prospective physicians (in randomised studies), or pre-intervention quality parameters (in non-randomised studies) [8]. Two reviewers (BPK and JS) independently reviewed each study against inclusion/exclusion criteria (additional file 2: Fig. S2). Studies were screened by titles and abstracts using DistillerSR online screening software. Full texts of 44 records were then reviewed, as well as an additional eight records that were identified from the study reference lists. All disputes were resolved by an expert third reviewer (LM). Researchers were blinded to each others’ decisions. A total of 21 studies were included for data extraction and final analysis (Fig. 2). One reviewer extracted data from the selected studies (BPK), with findings then reviewed by a second reviewer (JS). Disputes were resolved independently by the third reviewer (LM).

### Risk of bias, meta-analysis and narrative synthesis

Selected studies were categorised under ‘randomised’ and ‘non-randomised’, and risk of bias was assessed by two reviewers (BPK and JS) with disputes resolved by the third reviewer (LM). Randomised trials were assessed using the seven domains of the GRADE recommendations, and non-randomised studies were assessed using the seven domains of ROBINS-I criteria [18, 19].

All studies were initially assessed for clinical and methodological heterogeneity [20]. Four interventions were eligible to undergo meta-analysis in relation to five outcomes. As these were before-and-after studies without control groups, the ‘generic inverse variance method’ was used in pooling [21]. Review Manager 5.4 software was used in the meta-analysis and the effect measure was ‘risk difference’ (i.e. percentage of death certificates with each error). Statistical heterogeneity was assessed using the *I*-square statistic and chi-square test. When potential outliers were removed in dealing with statistical heterogeneity, sensitivity analyses were performed with and without excluded studies [22]. Robustness of the effect measures was explored further using a sensitivity analysis with both fixed and random effect assumptions [22]. Potential publication bias was explored with the generation of funnel plots.

The meta-analysis findings were imported through the ‘GRADEproGDT’ online tool. A ‘summary of findings’ table was prepared, and related narrative components added to the table [23]. The certainty assessments were done using eight criteria: study design, risk of bias, potential of publication bias, imprecision, inconsistency, indirectness, magnitude of effect, dose-response gradient and effect of plausible confounders [24]. Studies or sub-groups that were not included in the meta-analysis were included in a narrative synthesis of findings.

## Results

Within the 21 selected articles [13–15, 25–42], there were 24 distinct interventions, with one article describing four interventions across four countries [30]. In another, findings were stratified under two study populations [27]. Three were randomised controlled trials [13, 35, 37] and 21 were non-randomised interventions. Amongst the latter, one was a non-randomised controlled study [31] whilst the remainder were non-controlled before-after studies. Characteristics of the selected studies are shown in Table 1.

**Table 1:** Characteristics of selected studies

Study title used for analysis	Design	Country and target of intervention	Intervention group	Comparison group	Outcomes
Pain et al. 1996 [35]	Randomised controlled trial with one comparison group	UK; first year medical students.	92 students were allocated to a 15-min video plus the usual lecture; of these 71 saw the video and 85 took the test	93 students were allocated to the usual lecture; 91 took the test	1) Overall performance score out of 68: median (IQR). Intervention group: 42.0 (36.5–47.5); control group: 39.0 (35.0–45.0), *p* = 0.046; 2) Death certification score out of 44: median (IQR). Intervention group: 26 (22–30); control group: 25 (20–28), *p* = 0.066
Myers and Farquhar 1998 [34]	Quasi-experimental study with pre and post assessment of death certificates	Canada; Residents assigned to an internal medicine rotation	75-min seminar on proper completion of death certificates. 83 certificates completed after the intervention	146 death certificates completed before the intervention	1) At least one major error (mechanism of death only, improper sequence, competing causes): 48 (32.9%) pre- and 13 (15.7%) post-intervention, *p* = 0.01; 2) At least one minor error (absence of time intervals, abbreviations, mechanism followed by legitimate underlying cause of death (UCOD)): 123 (84.2%) pre- and 75 (90.4%) post-intervention, *p* = 0.19; 3) Mechanism of death only: 23 (15.8%) pre- and 4 (4.8%) post-intervention, *p* = 0.01; 4) Improper sequence: 23 (15.8%) pre- and 5 (6.0%) post-intervention, *p* = 0.03; 5) Competing causes: 11 (7.5%) pre- and 7 (8.4%) post-intervention, *p* = 0.81; 6) Absence of time interval: 101 (69.2%) pre- and 63 (75.9%) post-intervention, *p* = 0.28; 7) Abbreviations: 29 (19.9%) pre- and 15 (18.1%) post-intervention, *p* = 0.11; 8) Mechanism followed by legitimate UCOD: 67 (45.9%) pre- and 30 (36.1%) post-intervention, *p* = 0.15
Lakkireddy et al. 2007 [13]	Randomised interventional study with one comparison group	USA; 219 internal medicine residents from five teaching hospitals	Group I (45-min interactive workshop or ‘workshop group’, *n* = 105), 100 were available for analysis	Group II (printed handout or ‘print group’, *n* = 114), content was same as in Group I, 100 were available for analysis	1) Mid-America Heart Institute (MAHI) Death Certificate Score > 19: Group 1 20 (20%) pre- and 82 (82%) post-intervention, *p* < 0.001; Group II 18 (18%) pre- and 58 (58%) post-intervention, *p* < 0.001; Both groups 38 (19%) pre- and 140 (70%) post-intervention, *p* < 0.001; 2) MAHI Death Certificate mean Score: Group 1 (*n* = 100) 13.7 (+/− 5.9) pre- and 24.1 (+/− 4.8) post-intervention, *p* < 0.001; Group II (*n* = 100) 14.1 (+/− 4.6) pre- and 19.1 (+/− 5.4) post-intervention, *p* < 0.001, Both groups (*n* = 200) 13.9 (+/− 5.3) pre- and 21.6 (+/− 5.7) post-intervention, *p* < 0.001; 3) Correct identification of cause of death: Group 1 15 (15%) pre- and 91 (91%) post-intervention, *p* < 0.001; Group II 16 (16%) pre and 55 (55%) post *p* < 0.001; Both groups 31 (15.5%) pre- and 146 (84.5%) post-intervention, *p* < 0.001; 4) Erroneously identified cardiac death: Group 1 56 (56%) pre- and 6 (6%) post-intervention, *p* < 0.001; Group II 64 (64%) pre- and 43 (43%) post-intervention, *p* = 0.02; Both groups 120 (60%) pre- and 49 (24.5%) post-intervention, *p* < 0.001
Vilar and Perez-Mendez 2007 [41]	Quasi-experimental study with pre and post assessment	Spain; 166 Medical trainees from various medical specialties (family medicine, internal medicine, anaesthesiology, general surgery, critical care medicine) in seven teaching hospitals	90-min seminar on the proper completion of death certificates delivered as an interactive workshop, 166 death certificates filled after the intervention	166 death certificates filled before the intervention	1) At least one error: 71.1% pre- and 9.0% post-intervention, *p* < 0.0001; 2) Mechanism of death only: 71 (42.6%) pre- and 4 (2.4%) post-intervention, *p* < 0.0001; 3) Improper sequence: 31 (18.7%) pre- and 1 (0.6%) post-intervention, *p* < 0.0001; 4) Listing two causally unrelated, etiologically specific diseases as the cause of death: 10 (6%) pre- and 5 (3.0%) post-intervention, *p* = 0.290; 5) Abbreviations: 9 (5.4%) pre- and 5 (3.0%) post-intervention, *p* = 0.413; 6) Mechanism as UCOD: 22 (13.3%) pre- and 0 (0.0%) post-intervention, *p* < 0.0001; 7) Listing the cause of death in Part II: 46 (27.7%) pre- and 5 (3.0%) post-intervention, *p* < 0.0001
Degani et al. 2009 [14]	Quasi-experimental study with pre and post assessment	USA; All third-year medical students from Mercer University School of Medicine rotating at Medical Centre of Central Georgia	129 students were presented with a web-based tutorial lasting approximately 30 min, designed for self-study; 123 death certificates included in analysis	123 death certificates completed before the intervention	1) Modified version of MAHI Death Certificate Scoring system used (*n* = 123) with score out of 22; mean (SD): 11.75 (3.2) pre- and 18.85 (2.56) post-intervention. Mean difference 7.10 (3.86), *p* < 0.0001, *t* = 20.39
Pandya et al. 2009 [36]	Quasi-experimental study with pre and post assessment	India; 43 residents of target postgraduate disciplines at 550-bed teaching hospital	A structured 90-min presentation in one workshop followed by an interactive session. Second and third workshops included group activities. After the intervention 102 death certificates were assessed	96 death certificates from the pre-intervention period	1) Major: Unacceptable UCOD: 38 (39.6%) pre- and 25 (24.5%) post-intervention, *p* = 0.034; 2) Major: Mechanism only without UCOD: 13 (13.5%) pre- and 1 (1.0%) post-intervention, *p* = 0.001; 3) Major: Improper sequence: 24 (25.0%) pre- and 6 (5.9%) post-intervention, *p* = 0.0004; 4) Major: Competing causes: 37 (38.5%) pre- and 26 (25.5%) post-intervention, *p* = 0.069; 5) Minor: Absence of time interval: 28 (29.2%) pre- and 28 (27.5%) post-intervention, *p* = 0.91; 6) Minor: Abbreviations: 21 (21.9%) pre- and 34 (33.3%) post-intervention, *p* = 0.1; 7) Minor: Mechanism followed by legitimate UCOD: 13 (13.5%) pre- and 8 (7.8%) post-intervention, *p* = 0.28
Pieterse et al. 2009 [37]	Randomised interventional study with one comparison group	South Africa; 24 medical interns who had completed at least 6 months of their internship at an academic tertiary hospital	Death certification educational intervention consisting of a 45-min didactic teaching session and an educational handout (i.e. written guide). 13 were in the group	Written guide only. 11 were in the group	1) Score out of 30 for avoiding minor and major errors; mean (SD): Group 1 11.8 (1.8) pre- and 24.5 (1.0) post-intervention, *p* < 0.001; Group II 15.5 (1.5) pre- and 25.3 (1.1) post-intervention, *p* < 0.001; 2) Score out of 30: Group 1 15% pre- and 85% post-intervention, *p* = 0.004; Group II 9% pre and 91% post-intervention, *p* = 0.004; 3) Major: Mechanism only: Group 1 69% pre- and 15% post-intervention, *p* = 0.016; Group II 37% pre- and 27% post-intervention, *p* = 1.000; 4) Major: Improper sequence: Group 1 54% pre- and 0% post-intervention, *p* = 0.016; Group II 36% pre- and 36% post-intervention, *p* = 1.000; 5) Major: Competing causes: Group 1 69% pre- and 8% post-intervention, *p* = 0.008; Group II 73% pre- and 9% post-intervention, *p* = 0.039; 6) Minor: Absence of time interval: Group 1 77% pre- and 23% post-intervention, *p* = 0.016; Group II 64% pre- and 18% post-intervention, *p* = 0.063; 7) Minor: Abbreviations: Group 1 62% pre- and 8% post-intervention, *p* = 0.016; Group II 73% pre- and 9% post-intervention, *p* = 0.039
Hemans-Henry, Greene and Koppaka 2012 [31]	Non-randomised experimental study with one comparison group	USA; postgraduate year 1 (PGY1) internal medicine and general surgery residents (*n* = 114) and postgraduate year 2 (PGY2) internal medicine, emergency medicine, and general surgery residents (*n* = 113)	PGY1 residents completed a pre-test, e-learning course, post-test, and course evaluation. 59 completed all evaluations	74 PGY2 residents completed the same pre-test	The test consisted of 10 multiple-choice questions. The PGY1 and PGY2 average pre-test scores were comparable (59% and 61%, respectively). The average PGY1 post-test score was higher than both the average PGY1 pre-test score (72% vs 59%, respectively; *p* = 0.01); and the average PGY2 pre-test score (72% vs 61%, respectively; *p* = 0.001)
Walker et al. 2012 [15]	Quasi-experimental study with pre and post assessment	Fiji; Medical students in their final year who were undertaking their final week of education at the university	WHO training tool plus access to the online certification module. Participants completed the death certification module in the Fiji School of Medicine computer laboratory. 13 case vignettes were used in the post-test assessment. Responses of 23 participants were included	13 case vignettes were used in the pre-test assessment. Responses of 23 participants were included.	1). Quality index score and % were used (total score 15 per certificate × 13 certificates = 195; lower is better). Pre-test (*n* = 23) mean: 57.22 (22.91); post-test (*n* = 23) mean: 30.30 (11.66); mean change in quality index 26.91 (11.25); individual scores available and SD can be calculated; 2) Mean error rate: 33.14% pre- and 20.27% post-test; 3) Abbreviations: 19.40% improvement between pre- and post-test; 4) Reporting a legitimate sequence of events in Part I: 19.06% improvement; 5) Reporting only one cause per line: 18.06% improvement; 6) Reporting a disease and not a mode of death: 17.3% improvement; 7) Legibility: 1.67% improvement
Ali and Hamadeh 2013 [26]	Quasi-experimental study with pre and post assessment	Bahrain; 27 secondary healthcare physicians	Interactive workshop. Post-workshop death certificates were used, with each participant (*n* = 27) completing one certificate	Pre-workshop death certificates, with each participant (*n* = 27) completing one certificate	1) Listing mechanism without underlying disease: 2 (7.4%) pre- and 0 (0.0%) post-intervention, *p* = 0.491; 2) Improper sequence 1 (3.7%) pre- and 2 (7.4%) post-intervention, *p* = 1.0; 3) Listing two causally unrelated, etiologically specific diseases as the cause of death: 3 (11.1%) pre- and 0 (0.0%) post-intervention, *p* = 0.236; 4) Listing mechanism of death followed by proper UCOD: 18 (66.7%) pre- and 9 (33.3%) post-intervention, *p* = 0.009; 5) Listing the cause of death as one of the other significant conditions contributing to the death but not causally related to the immediate cause of death: 1 (3.7%) pre- and 0 (0.0%) post-intervention, *p* = 1.0; 6) Abbreviations 0 (0.0%) pre- and 0 (0.0%) post-intervention; 7) No error: 2 (7.4%) pre- and 16 (59.3%) post-intervention, *p* < 0.001
Azim et al. 2014 [28]	Quasi-experimental study with pre- and post-assessment (described as an observational study: audit- intervention and a re-audit)	India; 12 resident doctors undergoing their subspecialty training in critical care medicine	Educational intervention programme consisting of a lecture followed by an interactive session. 75 death certificates post-intervention were audited	75 pre-intervention death certificates	1) Major error: Unacceptable UCOD: 74 (98.6%) pre- and 31 (41.3%) post-intervention, *p* = 0.001; 2) Major: Mechanism only: 45 (60%) pre- and 11 (14.6%) post-intervention, *p* = 0.001; 3) Major: Improper sequence: 67 (89.3%) pre- and 27 (36.0%) post-intervention, *p* = 0.001; 4) Major: Competing causes: 66 (88.0%) pre- and 10 (13.3%) post-intervention, *p* = 0.001; 5) Minor: Absence of time interval: 75 (100.0%) pre- and 17 (22.6%) post-intervention, *p* = 0.001; 6) Minor: Abbreviations: 67 (86.3%) pre- and 22 (29.3%) post-intervention, *p* = 0.001; 7) Minor: Mechanism followed by legitimate UCOD: 12 (16.0%) pre- and 7 (6.6%) post-intervention, *p* = 0.55
Alonso-Sardon et al. 2015 [27]	Quasi-experimental study with pre- and post-assessment	Spain; 308 sixth year medical students	A formative intervention that included a five-hour on-site seminar-workshop, consisting of both theoretical and practical parts. Five completed death certificates were selected for comparison	Five death certificates filled before the intervention were selected for comparison	1) Major indexes consisted of assessment of underlying, intermediate and immediate causes; 2) Minor index: Mechanisms of death instead of causes; 3) Minor index: Inappropriate and vague terms; 4) Minor index: Abbreviations; 5) Minor index: Existence of multiple UCODs; 6) Minor index: Capital letters
		Spain; 62 practising family doctors and interns	A formative intervention including a five-hour on-site seminar-workshop with two parts; theoretical and practical. Five completed death certificates were selected for comparison	Five death certificates completed before the intervention were selected for comparison	1) Major indexes consisted of assessment of underlying, intermediate and immediate causes; 2) Minor index: Mechanisms of death instead of causes; 3) Minor index: Inappropriate and vague terms; 4). Minor index: Abbreviations; 5) Minor index: Existence of multiple UCODs; 6) Minor index: Capital letters
Miki et al. 2018 [32]	Quasi-experimental study with pre and post assessment	Peru; Doctors received either 1. an online intervention; or 2. an online intervention and a training intervention	1.’Online intervention’ - one hour on the online system (SINADEF) (900 death certificates) 2. ‘Online and training intervention’ - one hour on SINADEF and one-hour training on certification of cause of death (900 death certificates)	Pre intervention’ 300 pre-intervention death certificates	1): Major: Multiple causes per line: 1. Pre: 2.0%; 2. Online: 1.3%; 3. Online and training: 0.6%, *p* > 0.05; 2) Major: Absence of time interval: 1. Pre: 96.0%; 2. Online: 47.1%; 3. Online and training: 30.0%, *p* < 0.01; 3) Major: Incorrect sequence of events leading to death: 1. Pre: 40.3%; 2. Post: 25.9%, *p* < 0.05; 3. Online and training: 17.9%, *p* < 0.01; 4) Major: Ill-defined condition entered as UCOD: 1. Pre: 52.0%; 2. Post: 45.4%; 3. Online and training: 38.9%, *p* < 0.01; 5) Minor: Presence of blank lines within the sequence of events: 1. Pre: 11.3%; 2. Post: 0.2%; 3. Online and training: 0.3%, *p* < 0.01; 6) Minor: Abbreviations 1. Pre: 11.7%; 2. Post: 4.6%; 3. Online and training: 4.1%, *p* < 0.01); 7) Minor: Additional errors on the certificate: 1. Pre: 32.3%; 2. Post: 26.6%, *p* > 0.05; 3. Online and training: 21.0%, *p* < 0.01
Sudharson et al. 2019 [40]	Quasi-experimental study (described as a cross sectional study)	India; Teaching faculty post-graduates, junior residents and interns (who have completed medicine and surgery postings) (*n* = 80)	Lecture. Death certificates completed post-intervention based on a case scenario (*n* = 80)	Death certificates completed pre-intervention based on a case scenario (*n* = 80)	1) Major: Incorrect sequence of events: 48 (60.0%) pre- and 3 (3.75%) post-intervention; 2) Major: Unrelated causal events in sequence 6 (7.5%) pre- and 0 (0.0%) post-intervention; 3) Major: At least 1 major error: 51 (63.75%) pre- and 3 (3.75%) post-intervention; 4) Minor: Missing time interval: 68 (85.0%) pre- and 0 (0.0%) post-intervention; 5) Minor: Mechanism followed by legitimate UCOD: 66 (82.5%) pre- and 1 (1.25%) post-intervention; 6) Minor: Abbreviations: 18 (22.5%) pre- and 0 (0.0%) post-intervention; 7) Minor: At least 1 minor error: 78 (97.5%) pre- and 1 (1.25%) post-intervention
Hart et al. 2020 [30]	Comparison paper comparing multiple countries; ll quasi experimental studies with pre- and post-assessment	PNG; Physicians	Direct training of physicians on completion of death certificates. 378 post-training MCCODs	948 baseline MCCODs	1) Major: Multiple causes per line: 16.3% pre- and 7.9% post-intervention; 2) Major: Incorrect sequence: 41.7% pre- and 20.3% post-intervention; 3) Major: Illegible handwriting: 4.3% pre- and 1.6% post-intervention; 4) Major: Ill-defined cause as UCOD: 39.1% pre- and 18.7% post-intervention; 5) Major: Additional information on neoplasm not available: 4.5% pre; and 2.3% post-intervention; 6) At least one major error: 55.6% pre- and 30.7% post-intervention; 7) Minor: Abbreviations: 19.8% pre- and 5.4% post-intervention; 8) Minor: Absence of time interval: 74.7% pre- and 42.3% post-intervention; 9) Minor: Additional errors on the certificate: 5.3% pre- and 5.1% post-intervention; 10) At least one error: 86.4% pre- and 60.6% post-intervention
		Philippines; Physicians	Training of trainers and then direct training. 959 post-training MCCODs	975 baseline MCCODs	1) Major: Multiple causes per line: 21.2% pre- and 6.0% post-intervention; 2) Major: Incorrect sequence: 27.1% pre- and 12.4% post-intervention; 3) Major: Illegible handwriting: 0.3% pre- and 1.1% post-intervention; 4) Major: Ill-defined cause as UCOD: 28.6% pre- and 15.5% post-intervention; 5) Major: Additional information on external causes not available: 4.8% pre- and 1.2% post-intervention; 6) Major: Additional information on neoplasm not available: 2.3% pre- and 1.9% post-intervention; 7) At least one major error: 41.6% pre- and 22.6% post-intervention; 8) Minor: Abbreviations: 7.1% pre- and 0.8% post-intervention; 9) Minor: Absence of time interval: 37.4% pre- and 23.7% post-intervention; 10) Minor: Additional errors on the certificate: 5.3% pre- and 1.1% post-intervention; 11) At least one error: 72.9% pre- and 43.6% post-intervention
		Myanmar; Physicians	Training of trainers and then direct training. 600 post-training MCCODs assessed	595 baseline MCCODs assessed	1) Major: Multiple causes per line: 24.4% pre- and 10.8% post-intervention; 2) Major: Incorrect sequence: 7.9% pre- and 5.8% post-intervention; 3) Major: Illegible handwriting: 4.2% pre- and 2.8% post-intervention; 4) Major: Ill-defined cause as UCOD: 44.5% pre- and 32.7% post-intervention; 5) Major: Additional information on neoplasm not available: 1.4% pre- and 0.3% post-intervention; 6) At least one major error: 63.2% pre- and 44.8% post-intervention; 7) Minor: Presence of blank lines within the sequence of events: 0.2% pre- and 0.3% post-intervention; 8)Minor: Abbreviations: 50.8% pre- and 31.0% post-intervention; 9) Minor: Absence of time interval: 93.4% pre- and 65.3% post-intervention; 10) Minor: Additional errors on the certificate: 1.6% pre- and 0.7% post-intervention; 11) At least one error: 99.8% pre- and 74.8% post-intervention
		Sri Lanka; Physicians	Training of trainers and then direct training. 558 post-training MCCODs assessed	517 baseline MCCODs assessed	1) Major: Multiple causes per line: 38.9% pre- and 20.8% post-intervention; 2) Major: Incorrect sequence: 37.1% pre- and 17.0% post-intervention; 3) Major: Illegible handwriting: 0.6% pre- and 0.0% post-intervention; 4) Major: Ill-defined cause as UCOD: 4.4% pre- and 10.6% post-intervention; 5) Major: Additional information on neoplasm not available: 4.3% pre- and 0.5% post-intervention; 6) At least one major error: 58.8% pre- and 37.5% post-intervention; 7) Minor: Presence of blank lines within the sequence of events: 2.1% pre- and 2.7% post-intervention; 8) Minor: Abbreviations: 36.0% pre- and 20.3% post-intervention; 9) Minor: Absence of time interval: 87.0% pre- and 53.2% post-intervention; 10) Minor: Additional errors on the certificate: 2.9% pre- and 0.2% post-intervention; 11) At least one error: 95.4% pre- and 68.5% post-intervention
Wood, Weinberg and Weinberg 2020 [42]	Quasi-experimental study with pre, immediate-post and 2-month-post assessment	Canada; 63 residents and nine staff physicians participated in the pre-survey; 67 residents and eight staff in the immediate-post survey; 18 residents and six staff in the 2-month-post survey	60-min didactic session with case scenarios at grand rounds. 372 mock death certificates completed at immediate-post survey and 103 at 2-month-post survey	351 mock death certificates completed pre-intervention	1) Mechanism of death used as underlying cause of death: Error Occurrence (EO) Rate (%): 17 pre-; 1 immediate-post; 3 at 2 months, *p* < 0.05; 2) Absence of UCOD: EO Rate: 15 pre-; 2 immediate-post; 10 at 2 months, *p* < 0.05; 3) Incorrect manner of death recorded: EO Rate: 23 pre-; 2 immediate-post; 2 at 2 months, *p* < 0.05; 4) Abbreviations: EO Rate: 29 pre-; 26 immediate-post, *p* > 0.05; 5 at 2 months, *p* < 0.05; 5) Signs and symptoms listed: 1 pre-; 2 immediate-post; 0 at 2 months, *p* > 0.05; 6) Illogical sequence: 4 pre-; 2 immediate-post; 4 at 2 months, *p* > 0.05; 7) UCOD not in last line: 23 pre-; 5 immediate-post; 7 at 2 months, *p* < 0.05; 8) Part 2 items listed in part 1 (all errors preceding this in the row): 13 pre-; 3 immediate-post; 11 at 2 months, *p* < 0.05; 9) Listing medical conditions: 8 pre-; 0 immediate-post; 0 at 2 months, *p* < 0.05; 10) Part 1 items listed in Part 2: 14 pre-; 4 immediate-post; 2 at 2 months, *p* < 0.05; 11) Incorrect manner of death recorded: 23 pre-; 2 immediate-post; 2 at 2 months, *p* < 0.05; 12) More than once condition per line in Part 1: 1 pre-; 1 immediate-post; 1 at 2 months, *p* > 0.05
Abos et al. 2006 [25]	Quasi-experimental study with pre- and post-assessment	Spain; Group of 135 physicians assigned to practice in the reformed network of primary care	90-min seminar (BEDTAR programme); post-intervention assessment of 3 cases	Pre-intervention performance	1) Error item ‘immediate cause’ in relation to each case; 2) Error item ‘cardiopulmonary arrest’ in relation to each case; 3) Error item ‘intermediate cause’ in relation to each case; 4) Error item ‘root cause’ in relation to each case available; 5) Error item ‘double fundamental cause’ in relation to each case; 6) Error item ‘Other processes’ in relation to each case; 7) Error item ‘Abbreviations’ in relation to each case; 8) Error item ‘Legible letter’ in relation to each case; 9) Error item ‘logical sequence’ in relation to each case; 10) Error item ‘use all information’ in relation to each case; 11) Error item ‘Invention’ in relation to each case; 12) Error item ‘Poor defined entity’ in relation to each case; 13) Error item ‘Use of lowercase’ in relation to each case
Canelo and Gonzalez 1995 [29]	Quasi-experimental study with pre- and post-assessment	Spain; 173 sixth year medical students	Seminar; six post-intervention death certificates completed by each participant	Six pre-intervention death certificates completed by each participant	1) Basic or fundamental cause is correct: 937 (90.26%) pre- and 1012 (97.49%) post-intervention; 2) Logical sequence is correct: 683 (65.79%) pre and 906 (87.28%) post-intervention; 3) Various basic causes of death are correct: 981 (94.50%) pre- and 1027 (98.94%) post-intervention; 4) Mechanisms/cause of death is correct: 879 (84.68%) in pre and 1004 (96.72%) in post; 5) No imprecise terms: 1026 (98.84%) pre- and 1033 (99.51%) post-intervention; 6) No Abbreviations or acronyms: 882 (84.97%) pre- and 1031 (99.32%) post-intervention; 7) Legible and lowercase: 491 (47.30%) pre- and 999 (96.24%) post-intervention
Selinger, Ellis and Harrigton 2007 [38]	Quasi-experimental study with pre- and post-assessment (described as a clinical audit)	England; Senior house officers (SHOs), staff grades, specialist registrars and consultants	Education was in three forms: (1) Presentation of the findings of the pre-assessment during a clinical governance meeting; (2) Each doctor was given individualised performance data and (3) the topic was highlighted during the induction of new doctors. Post-intervention, 85 case notes were assessed	140 case notes	1) Consultants’ name not given: 48.6% pre- and 18.0% post-intervention; 2) At least one mistake or omission: 58.6% pre- and 20.0% post-intervention; 3) Completed by doctors who did not meet the requirements of being involved in the patient’s care: 13.6% pre- and 2.4% post-intervention, *p* = 0.01
Myers and Eden 2007 [33]	Quasi-experimental study with pre- and post-assessment	Canada; 25 family physicians.	Half-day workshop with case scenarios; 16 completed the post-test	21 completed the pre-test	1) Decline in use of mechanisms of death as the UCOD; 2) Increased use of more specific diseases as the UCOD; 3) More knowledgeable about not using old age as a cause of death
Suarez et al. 1998 [39]	Quasi-experimental study with pre- and post-assessment	Spain; Medical students, interns and trainees in family and community medicine	120-min teaching programme. 472 post-intervention exercises	472 pre-intervention exercises	1) Correct immediate cause: 89.8% pre- and 98.5% post-intervention; 2) Correct intermediate cause: 78.2% pre- and 97.7% post-intervention; 3) Correct initial or fundamental cause: 83.5% pre- and 97.9% post-intervention; 4) Correct other processes: 91.3% pre- and 94.1% post-intervention; 5) Correct basic cause of death: 78.4% pre- and 97.2% post-intervention; 6) Legible: 98.1% pre- and 98.5% post-intervention; 7) Logical sequence: 97.9% pre- and 99.8% post-intervention; 8) No abbreviations or acronyms: 80.9% pre- and 82.6% post-intervention; 9) No omission of diseases: 82.6% pre- and 91.3% post-intervention; 10) Absence of causes not described: 88.1% pre- and 97.0% post-intervention; 11) Correct causal sequence: 82.0% pre- and 98.7% post-intervention

### Study populations, interventions and outcomes

In seven interventions, the study populations consisted of medical students [14, 15, 27, 29, 35, 39, 41]. These medical students were comprised of first year students (UK) [35], medical trainees in teaching hospitals (Spain) [41], third year students (USA) [14] and final year students (Fiji and Spain) [15, 29]. Generally, however, the study populations were physicians or doctors, and referred to as residents (Canada, USA, India) [13, 28, 34, 36], medical interns (South Africa, Spain) [37, 39], postgraduates (USA, India) [31, 36, 40], secondary healthcare physicians (Bahrain) [26], family doctors (Spain, Canada) [27, 33, 39] or Senior House Officers (England) [38].

Seminars, interactive workshops, teaching programmes and training sessions were the most common terms used in introducing the interventions. These ranged in duration from 45 min [13] to 5 h [27], and some interventions included subsequent sessions on additional days [36]. Other descriptions included ‘training of trainers’ (Philippines, Myanmar, Sri Lanka) [30], a video (UK) [35] and web-based or online training (USA, Fiji) [14, 15, 31]. In Peru, training was complementary to an online death certification system [32].

For the majority of interventions, a comparison of certification errors pre- and post-intervention was used as the measure of impact, although some studies developed a special knowledge test or used a quality index. These included the Mid-America-Heart Institute (MAHI) Death-Certificate-Scoring System (two interventions) [13, 14], knowledge assessment tests developed by the investigators (three interventions) [31, 35, 37], and quality indexes providing numerical scores based on ICD volume 2 best-practice certification guidelines [15].

### Risk of bias assessments

**Fig. 3:**
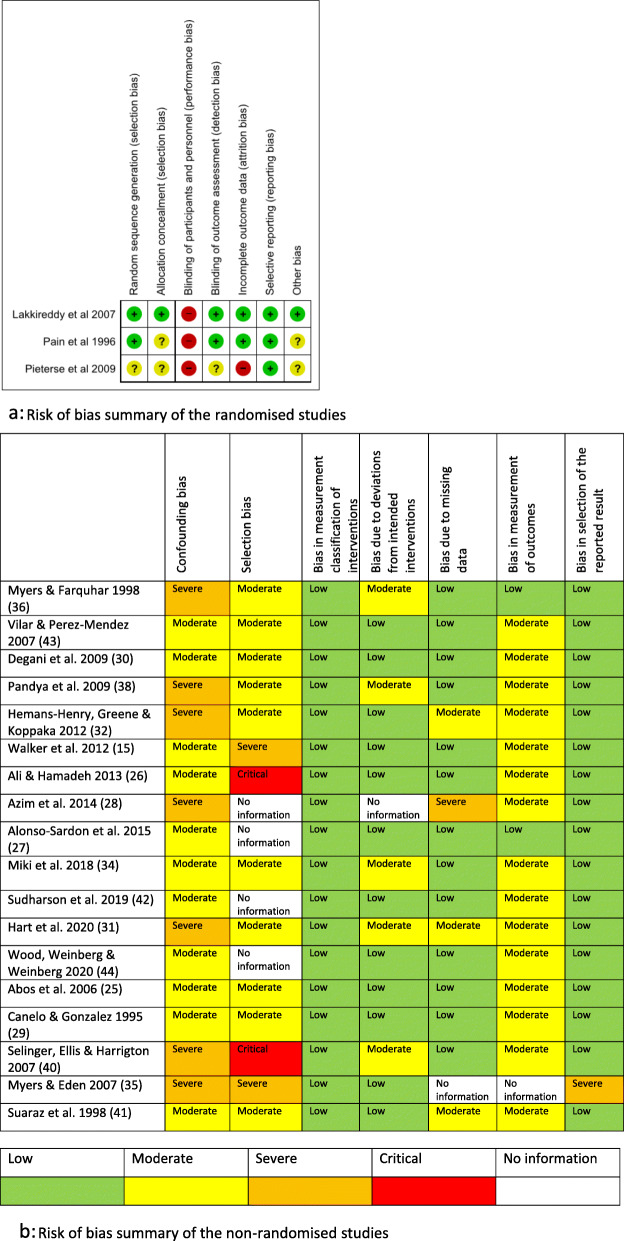
**a** Risk of bias summary of the randomised studies. **b** Risk of bias summary of the non-randomised studies

The risk of bias assessments for the randomised studies [13, 35, 37] are shown in Fig. 3a and in Fig. 3b for the non-randomised studies.

For all randomised studies, ‘blinding of participants and personnel’ was assessed as high-risk given the difficulty of maintaining blinding for training interventions. All three studies had pre-determined outcomes and were rated low risk for ‘selective reporting’.

All but one study were before-after studies without a separate control group. Due to the method of recruitment, none of the studies was characterised as low-risk in relation to confounding and selection bias. However, since the intervention periods were clearly defined, all studies were characterised as low-risk for ‘bias in measurement classification of interventions’.

### Meta-analysis

Since the interventions targeting medical students were found to be clinically heterogenous, potential meta-analyses were restricted to those targeting physicians. In anticipation of substantial methodological heterogeneity, the meta-analysis was planned separately for non-randomised studies. Findings of the studies and sub-groups initially entered to the meta-analysis are summarised in additional file 3: Tables S1-S5.

As the initial meta-analyses showed statistical heterogeneity, sensitivity analyses were performed after excluding a potential outlier in each comparison, with both fixed and random effect assumptions (Table 2). Except for ‘ill-defined underlying cause of death’ [43], the direction and significance of the estimates did not change with these sensitivity analyses.

**Table 2:** Sensitivity analysis of the pooled estimates

	Risk difference (95% CI) with fixed effect assumptions	Risk difference (95% CI) with random effect assumptions
All entered studies		
Improper sequence, *I*^2^ = 96%	0.11 (0.09 to 0.13)*	0.14 (0.05 to 0.24)*
Presence of abbreviations, *I*^2^ = 93%	0.09 (0.08 to 0.11)*	0.14 (0.07 to 0.20)*
No disease-time interval, *I*^2^ = 95%	0.27 (0.24 to 0.29)*	0.28 (0.18 to 0.38)*
Multiple causes in one line, *I*^2^ = 75%	0.13 (0.11 to 0.15)*	0.14 (0.10 to 0.17)*
Ill-defined UCOD, *I*^2^ = 97%	0.06 (0.04 to 0.08)*	0.10 (− 0.03 to 0.22)
After excluding the outliers		
Improper sequence, *I*^2^ = 64%	0.18 (0.15 to 0.20)*	0.18 (0.14 to 0.23)*
Presence of abbreviations, *I*^2^ = 21%	0.16 (0.13 to 0.18)*	0.16 (0.13 to 0.19)*
No disease-time interval, *I*^2^ = 0%	0.33 (0.30 to 0.36)*	0.33 (0.30 to 0.36)*
Multiple causes in one line, *I*^2^ = 0%	0.15 (0.13 to 0.17)*	0.15 (0.13 to 0.17)*
Ill-defined UCOD, *I*^2^ = 70%	0.15 (0.12 to 0.17)*	0.15 (0.10 to 0.20)*

*Statistically significant

**Fig. 4:**
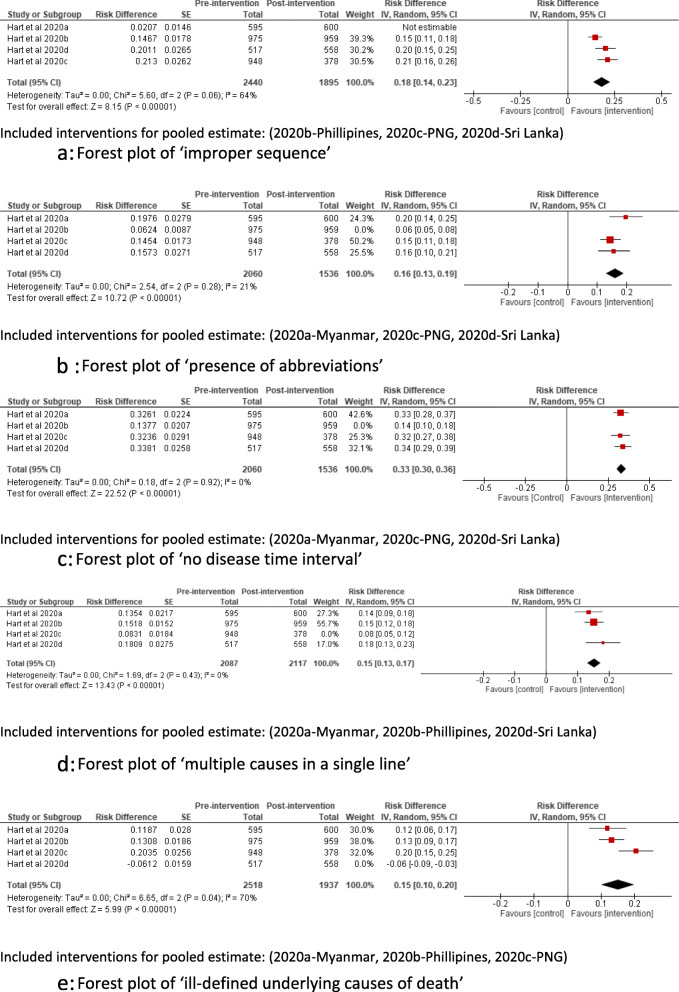
**a** Forest plot of ‘improper sequence’. **b** Forest plot of ‘presence of abbreviations’. **c** Forest plot of ‘no disease time interval’. **d** Forest plot of ‘multiple causes in a single line’. **e** Forest plot of ‘ill-defined underlying causes of death’

The forest plots of the five outcomes (i.e. after excluding the outliers) included in the meta-analyses are shown in Fig. 4a–e. Three interventions were included in each meta-analysis [30].

The lowest pooled risk difference (15%) was observed for ‘multiple causes per line’ and ‘ill-defined underlying cause of death’ whereas the highest was for ‘no disease time interval’ (33%).

**Fig. 5:**
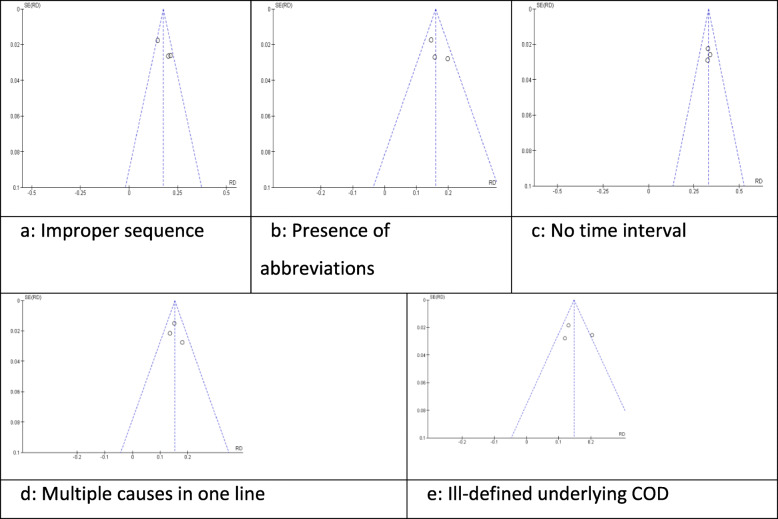
**a** – **e** Funnel plots of the pooled estimates

Funnel plots exploring potential publication bias are shown in Fig. 5a–e.

All funnel plots were generally symmetrical. A cautious interpretation of these is included in the “Discussion” section.

In the ‘summary of findings’ table (Table 3), the certainty assessments of these five outcomes are presented. ‘Moderate certainty’ was assigned to four outcomes and ‘low certainty’ to one. Findings of related additional studies have also been summarised as comments in Table 3.

**Table 3:** Summary of findings

Impact of Medical Certification of Cause of Death (MCCOD) training interventions in improving the quality of MCCOD						
Patient or population: Physicians or prospective physicians Setting: Global Intervention: Generic academic training in training curricula Comparison: Pre-intervention parameters of MCCOD quality						
Outcomes	Anticipated absolute effects* (95% CI)		Risk difference (95% CI)	№ of certificates assessed (studies)	Certainty of the evidence (GRADE)	Comments regarding similar studies that did not meet the meta-analysis inclusion criteria
	Risk with pre-intervention	Risk with post-intervention				
No time interval	832 per 1000	**275 per 1000** (250 to 300)	**0.33** (0.30 to 0.36)	3596 (3 observational studies)	⨁⨁⨁◯ Moderate^a^	In one study in Canada, 83 and 146 death certificates were assessed with 69.2% and 75.9% error percentages. In one Indian study, the related percentages were 29.2% and 27.5%. In another two Indian studies with just 75 and 80 death certificate assessments, the percentages were 100% versus 22.6%, and 85% versus 0.0%, respectively
Presence of abbreviations	328 per 1000	**53 per 1000** (43 to 59)	**0.16** (0.13 to 0.18)	3596 (3 observational studies)	⨁⨁⨁◯ Moderate^a^	In the above Canadian study, the error percentages were 19.9% and 18.1%. In the three Indian studies, the related percentages were 21.9.% versus 33.3%; 86.3% versus 29.3%; and 22.5% versus 0.0%, respectively
Improper sequence	349 per 1000	**63 per 1000** (52 to 70)	**0.18** (0.15 to 0.20)	4335 (3 observational studies)	⨁⨁⨁◯ Moderate^a^	In the above Canadian study, the error percentages were 15.8% and 6%. In the three Indian studies, the related percentages were 25% versus 59%; 89.3% versus 36%; and 60% versus 3.75%, respectively
Multiple causes	265 per 1000	**40 per 1000** (34 to 45)	**0.15** (0.13 to 0.17)	4204 (3 observational studies)	⨁⨁⨁◯ Moderate ^a^	In one study in Papua New Guinea, the respective percentages were 16.3% and 7.9%
Ill-defined underlying cause of death	363 per 1000	**55 per 1000** (44 to 62)	**0.15** (0.12 to 0.17)	4455 (3 observational studies)	⨁⨁◯◯ Low^a,b^	In one Sri Lankan study, ill-defined underlying cause of death was observed to be higher post-intervention (10.6% versus 4.4%)

GRADE Working Group grades of evidence. High certainty: We are very confident that the true effect lies close to that of the estimate of the effect. Moderate certainty: We are moderately confident in the effect estimate: the true effect is likely to be close to the estimate of the effect, but there is a possibility that it is substantially different. Low certainty: Our confidence in the effect estimate is limited: the true effect may be substantially different from the estimate of the effect. Very low certainty: We have very little confidence in the effect estimate: the true effect is likely to be substantially different from the estimate of effect

*CI* Confidence interval

^a^Due to being non-randomised studies and since in some studies, pre- and as post-analyses were not done immediately close to the intervention; the bias due to confounding was marked as ‘serious’

^b^Funnel plot not fully symmetrical in one study that underwent meta-analysis

*The risk in the intervention group (and its 95% confidence interval) is based on the assumed risk in the comparison group and the relative effect of the intervention (and its 95% CI)

### Narrative synthesis of other findings

#### Findings of randomised studies

In two of the three randomised studies conducted on medical interns, overall scores improved with the intervention (*p* < 0.05) [13, 37]. In the third study, which was conducted on medical students, there was weak evidence for an improvement in the overall performance score (*p* = 0.046), as well as a ‘skill score’ (*p* = 0.066) [35]. In one study, ‘correct identification of the COD’ improved more in the intervention group (15% to 91%) compared to the control group (16% to 55%), and ‘erroneous identification of cardiac deaths’ decreased more with the intervention (56% to 6%) compared to the controls (64% to 43%) [13]. In a South African study, three errors (‘mechanism only’, ‘improper sequence’ and ‘absence of time interval’) were significantly reduced in the intervention group only, whereas ‘competing causes’ and ‘abbreviations’ were reduced in both groups [37].

#### Non-randomised study findings on medical students

Degani et al. (2009) showed improvements in the modified-MAHI score following the intervention (mean difference of 7.1; *p* < 0.0001) [14]. Vilar and Perez (2007) reported improvements in ‘at least one error’ (*p* < 0.0001), including ‘mechanism of death only’ (*p* < 0.0001), ‘improper sequence’ (*p* < 0.0001), ‘listing cause of death in Part 2’ (*p* < 0.0001) and ‘mechanism as UCOD’ (*p* < 0.0001) [41]. In the same study, two error types (‘abbreviations’ and ‘listing two causally related causes as COD’) did not show evidence of improvement (*p* = 0.413 and *p* = 0.290) [41]. In a Fijian study, training produced improvements of 1.67% to 19.4% in the following: ‘quality index score’, ‘average error rate’, ‘abbreviations’, ‘sequence’, ‘one cause per line’, ‘not reporting a mode of death’ and ‘legibility’ [15]. In two Spanish studies, the intervention improved performance in ‘sequence’, ‘cause of death’, ‘precision of terms’, ‘abbreviations’ and ‘legibility’ [29, 39].

#### Other comparisons

Case-wise comparisons with a set of errors were conducted in two studies [25, 27]. Most errors decreased following the intervention. In one non-randomised controlled study, a custom performance score increased post-intervention [31]. One study in England explored ‘mentioning consultant’s name’ and ‘completion by a non-involved doctor’, both of which improved following the intervention [38]. In a Canadian study, ‘increased use of specific diseases as UCOD’ and ‘being more knowledgeable on not using conditions like ‘old age’’ improved in the intervention group [33]. ‘Competing causes’ were less common post-intervention in two Indian studies, with varying strength of evidence (*p* = 0.001 and *p* = 0.069) [28, 36], but not in a Canadian study (*p* = 0.81) [34]. ‘Mechanism of death followed by a legitimate UCOD’ showed non-significant reductions in three studies (45.9% to 36.1%, 13.5% to 7.8% and 16% to 6.6%) [28, 34, 36]. Other studies that assessed ‘presence of at least one-major error’ and ‘keeping blank lines’ in the sequence generally showed a reduction following the intervention [30, 34].

## Discussion

We conducted a systematic review of the impact of 24 selected interventions to improve the quality of MCCOD. Our meta-analysis suggests that selected training interventions significantly reduced error rates amongst participants, with moderate certainty (four outcomes), and low certainty (one outcome). Similarly, the findings of the narrative synthesis suggest a positive impact on both physicians and medical trainees. These findings highlight the feasibility and importance of strengthening the training of current and prospective physicians in correct MCCOD, which will in turn increase the quality and policy utility of data routinely produced by vital statistics systems in countries.

The systematic approach we followed distinguishes this study from the more common ‘narrative reviews’, whilst the meta-analysis provides pooled and precise estimates of training impact [44]. Rigorous heterogeneity and ‘certainty of evidence’ assessments were performed. To enable a better comparison of the quality of the selected studies, risk of bias assessments were performed using different criteria for randomised and non-randomised studies [18, 19]. Given the controversy surrounding conventional direct comparison methods for before-after studies in the literature—due to these methods’ non-independent nature [45]—less controversial ‘generic inverse variance methods’ were used in this review.

Irrespective of the study design (i.e. randomised or not) and population (i.e. physicians or medical students), training interventions were shown to reduce diagnostic errors, either in relative terms or due to an increase in scaled scores. Risk differences were used as pooled effect measures and typically suggested that certification errors decreased between 15 and 33% as a result of the training. Our findings also suggest that refresher trainings and regular dissemination of MCCOD quality assessment findings can further reduce diagnostic errors. However, due to the inherent limitations of using ‘absolute risk estimates’ like risk differences, we place greater emphasis on the direction of the effect measure and not on its size [46].

The pre-intervention percentages of all error categories selected for meta-analyses were below 51%, except for the category ‘absence of time intervals’, which ranged from 37 to 93% [30]. Based on post-intervention percentages, we therefore conclude that the intervention had a markedly favourable impact. For example, post-intervention errors were reduced to between 6.0 and 20.8% for ‘multiple causes in a single line’ and between 5.8 and 20.3% for ‘improper sequence’. For all interventions reviewed under the meta-analysis, post-training assessments were conducted between 6 months and 2 years after the intervention. Hence, the observed risk differences reflect the impact of the intervention over a longer time period, which is likely to be a more useful measure of the sustainability and effectiveness of training interventions than the more commonly used immediate post-training assessments.

The classification of errors into ‘minor’ or ‘major’ varies between studies. For example, ‘absence of time intervals’ was considered a major error in one study [32], but minor in several others [28, 30, 34, 36]. Some studies, although not all, classified ‘mechanism of death followed by a legitimate UCOD’ as an error [26, 28, 34, 36, 40]—furthermore, the scoring method and content of the assessment varied between studies [13, 14, 31, 35, 37]. Given this heterogeneity, it is important to focus on the patterns of individual errors and to be clear about how errors are defined before comparing results across studies.

Interestingly, we found greater variation across studies for post-intervention composite error indicators than for specific errors. Across the six interventions considered, post-intervention measures of ‘at least one major error’ ranged from 3.75 to 44.8% [30, 34, 40] whilst the fraction of cases with ‘at least one error’ ranged from 9 to 74.8% [30, 38, 41]. It is also interesting to note that doctors appeared to benefit less from the interventions compared to interns. This may in part reflect lower priority given by doctors to certification compared to patient management, possibly due to limited understanding of the public policy utility of data derived from individual death certificates.

In some studies, it is possible that a small proportion of post-intervention death certificates were actually completed by doctors who had not undergone training. This would have the effect of diluting the impact estimates of the training interventions. Further, constructing the causal sequence on the death certificate may involve a degree of public health and epidemiological consideration, in addition to clinical reasoning, which may be challenging for some doctors to incorporate into the certification process. This could explain the general lower improvement scores reported for the causal sequence. Finally, correct certification practices are heavily dependent on the attitudes of doctors towards the process, as well as the level of monitoring, accountability and feedback related to their certification performance.

Most interventions were conducted as interactive workshops that enabled participants to undergo ‘on-the-spot’ training [13, 25–30, 33, 34, 36, 37, 41]. There is a paucity of studies with control groups that compare different interventions. One study concluded that a ‘face-to-face’ intervention was more effective than ‘printed instructions’ [13]. However, another concluded that an added ‘teaching session’ did not improve performance compared to an ‘education handout’, although both strategies were independently effective [13, 37]. More research is required to test the relative effectiveness of training methods, such as online interventions, compared to those requiring face-to-face interaction.

Our analysis suggests several cost-effective options for improving the quality of medical certification. To the extent that individual-level training of doctors in correct medical certification is costly, strengthening the curricula in medical schools designed to teach medical students how to correctly certify causes of death, and ensuring that these curricula are universally applied, is likely to be the most economical and sustainable way to improve the quality of medical certification. How and when this training is applied prior to completion of medical training is likely to vary from one context to another and will depend on local requirements for internship training. Training smaller groups of physicians as master trainers in medical certification and subsequently rolling out the training in provincial and district hospitals is likely to be an effective and economical interim measure to improve certification accuracy, as has been demonstrated in a number of countries [30].

In some countries, electronic death certification has been used as a means to standardise and improve the quality of cause of death data [32]. Electronic death certification can be helpful in avoiding certain errors such as illegible handwriting and reporting multiple causes on a single line (by not allowing the certifier to report more than one condition per line) [47]. An electronic certification system can also generate pop-up messages to remind the certifier not to report modes of dying, or symptoms and signs, as the underlying cause. However, electronic certification cannot improve the accuracy of the causal sequence or alleviate the reporting of competing causes, unspecified neoplasms or non-reporting of external causes. Furthermore, whilst cause of death data entered in free text format could improve the quality of medical certification [48] when electronic certification is enhanced with suggested text options and ‘pick’ lists, this can lead to systematic errors in medical certification.

This review has several limitations. The studies examined in this review included a diverse range of participants and intervention methods and were conducted in various cultural settings. The duration and modality of the training interventions varied substantially across studies. Only three interventions were randomised, and due to the diversity in non-randomised studies, the potential influence of confounding factors on the quality parameters assessed cannot be excluded. These factors were, however, considered in risk of bias and heterogeneity assessments.

There is also considerable subjectivity in the assessment of some criteria, including ‘legibility’ and ‘incorrect sequence’ that could lead to bias in the assessments. Despite outcomes usually being pre-defined, adherence to risk-lowering strategies, such as ‘blinding the assessor’, was often not described [14, 15, 25, 26, 28–33, 36, 38–42]. Despite the inclusion of only three interventions, each meta-analysis included an adequate number of at least 1500 observations per group. Even though funnel plots were presented for gross exploration of publication bias, generally the interpretation of these are recommended for meta-analyses with more than 10 comparisons. Furthermore, little evidence is available on the appropriateness of funnel plots drawn with risk differences [49].

## Conclusions

Both pooled estimates and narrative findings demonstrate the effectiveness of training interventions in improving the accuracy of death certification. Meta-analyses revealed that these interventions are effective in reducing diagnostic errors, including ‘no time interval’, ‘using abbreviations’, ‘improper sequence’, ‘multiple causes per line’ with moderate certainty and ‘ill-defined underlying CoDs’ with ‘low certainty’. In general, ‘no time interval’ was observed to be the most common error, and ‘illegibility’ the least observed amongst pre-intervention errors. ‘No time interval’ appeared to be the error with most improvement following intervention, as evidenced by both the pooled and narrative findings.

Strategic investment in MCCOD training activities will enable long-term improvements in the quality of cause of death data in CRVS systems, thus improving the utility of these data for health policy. Whilst these findings strengthen the evidence base for improving the quality of MCCOD, more research is needed on the relative effectiveness of different training methods in different study populations. From the limited evidence thus far, our meta-analysis indicates that training doctors and interns in correct cause of death certification can increase the accuracy of certification and should be routinely implemented in all settings as a means of improving the quality of cause of death data.

## Supplementary Information

**Additional file 1: Figure S1.** Search strategy used in the review of literature.

**Additional file 2: Figure S2.** Selection criteria used in study selection.

**Additional file 3: Tables S1-S5.** Data used for meta-analysis.

## Data Availability

All data generated or analysed during this study are included in this published article (and its supplementary information files).
