# Supplementary material for: Effectiveness of training interventions to improve quality of medical certification of cause of death: systematic review and meta-analysis
Source: BMC Med. 2020 Dec 11;18:384. doi: 10.1186/s12916-020-01840-2 (PMC7728523; doi:10.1186/s12916-020-01840-2)
Supplement: Supplementary file 1 — Additional file 1: Figure S1. Search strategy used in the review of literature. [file 12916_2020_1840_MOESM1_ESM.docx]

**Figure 1: Search strategy used in the review of literature, included as a word file with the extension “docx”**

The following search terms were used in the CENTRAL database:

([mh "internet-based intervention"] OR "internet-based intervention" OR [mh "internet intervention"] OR "internet intervention" OR [mh "premedical education"] OR "premedical education" OR [mh "professional education"] OR "professional education" OR [mh "inservice training"] OR "inservice training" OR [mh "simulation training"] OR "simulation training" OR "intervention" OR "training" OR "staff training") AND ([mh "death certificates"] OR "death certificate" OR "medical certification of cause of death" OR "certification of cause of death" OR "certification of causes of death" OR "certification of cause of death" OR "death certification" OR "cause of death certification" OR "cause-of-death certification")

The following search terms were used in OVID MEDLINE and OVID EMBASE databases;

((internet-based intervention OR internet intervention OR premedical education or professional education OR in service training OR simulation training).af OR intervention.mp OR training.mp OR staff training.mp) AND ((death certificates).af OR death certificate.mp OR medical certification of cause of death.mp OR certification of cause of death.mp OR death certification.mp OR cause of death certification.mp OR cause-of-death certification.mp).
