# Supplementary material for: Effectiveness of training interventions to improve quality of medical certification of cause of death: systematic review and meta-analysis
Source: BMC Med. 2020 Dec 11;18:384. doi: 10.1186/s12916-020-01840-2 (PMC7728523; doi:10.1186/s12916-020-01840-2)
Supplement: Supplementary file 2 — Additional file 2: Figure S2. Selection criteria used in study selection. [file 12916_2020_1840_MOESM2_ESM.docx]

**Figure S2: Selection criteria used in study selection**

1. Does this study assess a novel (i.e. non-generic academic training in training curricula) training intervention to improve MCCOD?

- Yes (include)

- No (exclude)

- Unsure (neutral)

2. Are the study participants current or prospective physicians?

- Yes (include)

- No (exclude

- Unsure (neutral)

- N/A (exclude)

3. Do the study outcomes include change in errors in cause of death documentation and/or change in percentage of death codes that are garbage codes?

- Yes (include)

- No (exclude

- Unsure (neutral)

- N/A (exclude

4. Is the study a systematic review, RCT, quasi-experimental or observational study?

- Yes (incl)

- No (excl)

- Not sure (neutral)
