# Supplementary material for: Effectiveness of training interventions to improve quality of medical certification of cause of death: systematic review and meta-analysis
Source: BMC Med. 2020 Dec 11;18:384. doi: 10.1186/s12916-020-01840-2 (PMC7728523; doi:10.1186/s12916-020-01840-2)
Supplement: Supplementary file 3 — Additional file 3: Tables S1-S5. Data used for meta-analysis. [file 12916_2020_1840_MOESM3_ESM.docx]

**Table S1: Findings of studies or sub-groups entered for the meta-analysis of ‘improper sequence’**

| Study | Pre-training | | Post-training | | RD | SE |
| --- | --- | --- | --- | --- | --- | --- |
|  | Events | Total | Events | Total |  |  |
| Hart et al. 2020a | 47 | 595 | 35 | 600 | 0.0207 | 0.0146 |
| Hart et al. 2020b | 264 | 975 | 119 | 959 | 0.1467 | 0.0178 |
| Hart et al. 2020c | 395 | 948 | 77 | 378 | 0.2130 | 0.0262 |
| Hart et al. 2020d | 192 | 517 | 95 | 558 | 0.2011 | 0.0265 |

**Table S2: Findings of studies or sub-groups entered for the meta-analysis of ‘presence of abbreviations’**

| Study | Pre-training | | Post-training | | RD | SE |
| --- | --- | --- | --- | --- | --- | --- |
|  | Events | Total | Events | Total |  |  |
| Hart et al. 2020a | 302 | 595 | 186 | 600 | 0.1976 | 0.0279 |
| Hart et al. 2020b | 69 | 975 | 8 | 959 | 0.0624 | 0.0087 |
| Hart et al. 2020c | 188 | 948 | 20 | 378 | 0.1454 | 0.0173 |
| Hart et al. 2020d | 186 | 517 | 113 | 558 | 0.1573 | 0.0271 |

**Table S3: Findings of studies or sub-groups entered for the meta-analysis of ‘no- time interval**’

| Study | Pre-training | | Post-training | | RD | SE |
| --- | --- | --- | --- | --- | --- | --- |
|  | Events | Total | Events | Total |  |  |
| Hart et al. 2020a | 556 | 595 | 392 | 600 | 0.3261 | 0.0224 |
| Hart et al. 2020b | 365 | 975 | 227 | 959 | 0.1377 | 0.0207 |
| Hart et al. 2020c | 708 | 948 | 160 | 378 | 0.3236 | 0.0291 |
| Hart et al. 2020d | 450 | 517 | 297 | 558 | 0.3381 | 0.0258 |

**Table S4: Findings of studies or sub-groups entered for the meta-analysis of ‘multiple causes’**

| Study | Pre-training | | Post-training | | RD | SE |
| --- | --- | --- | --- | --- | --- | --- |
|  | Events | Total | Events | Total |  |  |
| Hart et al. 2020a | 145 | 595 | 65 | 600 | 0.1354 | 0.0217 |
| Hart et al. 2020b | 207 | 975 | 58 | 959 | 0.1518 | 0.0152 |
| Hart et al. 2020c | 154 | 948 | 30 | 378 | 0.0831 | 0.0184 |
| Hart et al. 2020d | 201 | 517 | 116 | 558 | 0.1809 | 0.0275 |

**Table S5: Findings of studies or sub-groups entered for the meta-analysis of ‘ill-defined underlying cause of death’**

| Study | Pre-training | | Post-training | | RD | SE |
| --- | --- | --- | --- | --- | --- | --- |
|  | Events | Total | Events | Total |  |  |
| Hart et al. 2020a | 265 | 595 | 196 | 600 | 0.1187 | 0.0280 |
| Hart et al. 2020b | 279 | 975 | 149 | 959 | 0.1308 | 0.0186 |
| Hart et al. 2020c | 371 | 948 | 71 | 378 | 0.2035 | 0.0256 |
| Hart et al. 2020d | 23 | 517 | 59 | 558 | -0.0612 | 0.0159 |
